# Lack of association between matrix metalloproteinase-1 gene rs1799750 polymorphism and osteoarthritis susceptibility: a meta-analysis

**DOI:** 10.1042/BSR20181960

**Published:** 2019-04-12

**Authors:** Lei Peng, Jie Bin, Yang-chao Ou, Li-xin Zhu, Ji-ping Lu

**Affiliations:** Department of Orthopedics, The Second Affiliated Hospital of Hunan Normal University, The NO. 921 Hospital of the People’s Liberation Army Joint Support Force, Changsha, Hunan, China

**Keywords:** gene, MMP-1, osteoarthritis, polymorphism

## Abstract

**Background.** A relationship between matrix metalloproteinase-1 (MMP-1)-1607 (rs1799750) gene polymorphism and osteoarthritis (OA) susceptibility was reported in the *Bioscience Reports* journal; however, these results were inconsistent. To evaluate the specific relationship, we used a meta-analysis study to clarify the controversy. **Methods.** The relevant articles were retrieved on 20 October 2018 from PubMed, Elsevier, Springer, Ebase (Ovid), and Google Scholar. The number of alleles and genotypes for MMP-1 was obtained. Odds ratios (ORs) and 95% confidence intervals (CIs) were used to estimate the association between MMP-1-1607 (rs1799750) 1G/2G promoter polymorphism and OA, while the Egger’s test was used to assess heterogeneity among studies and publication bias. All statistical analyses were conducted using STATA 12.0 software. **Results.** A total of six case–control studies covering 1133 cases and 1119 controls were included in the final meta-analysis. There was no significant association between MMP-1-1607 1G/2G promoter polymorphism and OA in all genetic models (2G versus 1G: OR = 1.12, 95% CI = 0.78–1.60; 1G/2G versus 1G/1G: OR  = 0.73, 95% CI = 0.32–1.67; 2G/2G versus 1G/1G: OR  =  1.31, 95% CI = 0.57–2.98; the recessive model: OR  =  1.23, 95% CI = 0.63-2.41; and the dominant model: OR  =  1.25, 95% CI = 0.79–1.97). We obtained similar results for the subgroup analysis using ethnicity and type of disease. **Conclusion.** We systematically investigated the association between MMP-1-1607 (rs1799750) 1G/2G polymorphism and OA susceptibility; however, the results show no correlation.

## Introduction

Osteoarthritis (OA) is a common degenerative joint disease resulting from focal loss of the protective cartilage layer at the ends of the bones [1]. The prevalence of OA is higher in women than men, especially after menopause [2,3]. Currently, OA occurs in 14% of adults aged 25 years and above [4] and is characterized by the loss of articular cartilage, which is accompanied by altered function of other synovial joint tissues [5]. The factors and genetic participation in mechanisms of OA, as well as OA etiology, are still unknown. Recently, genes have become more important in disease diagnosis, therapy, and susceptibility. Therefore, the analysis and identification of new genes associated with OA susceptibility is a vital and significant challenge. Early diagnosis is extremely important for OA prevention and management. OA is diagnosed using radiographic and medical history; however, these diagnostic methods are relatively poor for the early diagnosis of OA [2]. The degeneration and destruction of articular cartilage is an early change in the OA process.

A recently described single nucleotide polymorphism (SNP) in the promoter sequence of the matrix metalloproteinase-1 (MMP-1, collagenase-1) gene may play a significant role in controlling MMP-1 gene expression and is located on the long arm of chromosome 11 [6]. The two alleles of MMP-1-1607 (rs1799750) gene polymorphism (1G and 2G) are formed by an insertion/deletion of guanine at position -1607. There are many SNPs in the promoter region of the *MMP-1* gene [6]. MMP-1, an important member of the matrix metalloproteinase (MMPs) family, plays a key role in the degradation and destruction of articular cartilage and bone, and is closely associated with OA, periodontal disease, rheumatoid arthritis, and some tumors [7–9].

To date, many researchers have reported the relationship between MMP-1 gene polymorphism and OA susceptibility; however, the results are contradictory or lack strength owing to small sample sizes. Consequently, the present study aimed to clarify the association between the *MMP-1* gene and OA susceptibility.

## Materials and methods

### Publication search and standard setting

For this meta-analysis, we worked with the Meta-analysis Of Observational Studies in Epidemiology (MOOSE) group [10]. PubMed, Elsevier, Springer, Ebase (Ovid), and Google Scholar were searched (until 20 October 2018) using the terms: ‘matrix metalloproteinase 1’, ‘collagenase-1’, ‘MMP-1’, ‘polymorphism’, ‘gene’, ‘osteoarthritis’, and ‘OA’, as both medical subject heading (MeSH) terms and text words to find all papers that had the study investigated the association of MMP-1 1G/2G SNP with OA. A manual search was conducted to find unknown references to additional studies. English language restrictions were applied. Studies were selected if they satisfied the following criteria: (i) case–control study; (ii) sufficient published data for calculating the odds ratio (OR) and 95% confidence interval (CI); (iii) the association of MMP-1-1607 (rs1799750) 1G/2G polymorphism with OA [11]; and (iv) there was a matched Hardy–Weinberg equilibrium (HWE) in control cases.

### Data extraction and evaluation of study quality

Two investigators (L.P. and J.L.) independently selected eligible studies according to the inclusion criteria listed above. The following items were extracted: first author, publication year, country, ethnicity, genotype distributions, HWE, and case and control sizes. In any case of dispute, the two investigators checked the collected data and reached a consensus through discussion. Data were extracted independently by two investigators (L.P. and J.L.) and the quality of included studies was evaluated by two independent investigators (L.P. and J.L.) according to the Newcastle–Ottawa Scale (NOS) for case–control studies [12]. The NOS ranges between zero (worst) and nine stars (best). Any dispute was resolved by discussion and, if required, a third investigator (J.B.) decided the outcome.

### Statistical methods

Pearson’s *X^2^* tested the deviation of estimates from HWE in the control group according to genotype distributions. Crude ORs with their 95% CIs were estimated and used to assess the strength of association between MMP-1-1607 (rs1799750) 1G/2G polymorphism and OA. The pooled OR was calculated for allelic effect of 1G versus 2G, homozygote comparison of 2G/2G versus 1G/2G, heterozygote comparison of the recessive model (2G/2G versus 1G/1G + 1G/2G), and the dominant model (2G/2G + 1G/2G versus 1G/1G). The significance of the pooled OR was determined by the Z-test (*P*≤0.05).

Cochran’s *Q* statistic (*P*<0.10 indicated evidence of heterogeneity) assessed the heterogeneity among studies [13]. When significant heterogeneity (*P*<0.10) was achieved, the random-effects model was used to combine the effect sizes of the included studies; otherwise the fixed-effects model was adopted [14]. Subgroup analysis was performed using ethnicity and type of disease. In addition, sensitivity analyses were performed to identify individual study effects on pooled results and test the reliability of results. An estimate of potential publication bias was carried out using a Begg’s funnel plot and Egger’s regression test. All of the statistical analyses were conducted using STATA 12.0 software (Stata Corporation, College Station, TX, U.S.A.), and the *P* (*P*≤0.05 was considered significant) values were all two sided. Our study followed the PRISMA guidelines.

## Results

### Characteristics of included studies and search results

**Figure 1:**
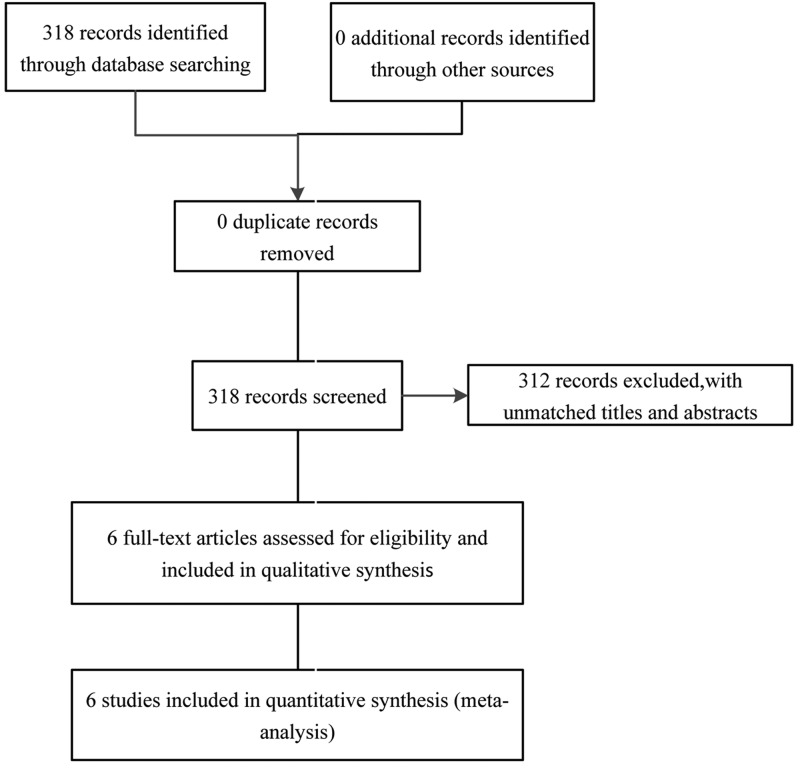
Studies identification diagram

Six case–control studies [15–20] (including 1133 cases and 1119 controls) met our inclusion criteria (Figure 1). The essential detailed characteristics and genotype distribution of eligible studies are summarized in Table 1. Among all studies, three involved Caucasians and three involved Asians. The distribution of genotypes in the controls was in agreement with HWE for all studies. The NOS table shows that the score of every study is higher than 6 points, which demonstrated the design of the case-control study was generally good (Table 1).

**Table 1:** Relevant studies investigating the relationship between MMP-1-1607 (rs1799750) 1G/2G polymorphisms and OA

Author	Year	Country	Ethnicity	Type of disease	Study design	Sample size of case/control	Genotyping methods	HWE among controls	Results
Luo et al.	2015	China	Asian	TMJ OA	Case–control	206/185	PCR-RFLP	0.58	*
Lepetsos	2014	Greece	Caucasian	Knee OA	Case–control	155/139	PCR-RFLP	0.06	NO
Yang et al.	2015	China	Asian	Knee OA	Case–control	207/207	PCR-RFLP	0.97	NO
Abd-Allah et al.	2012	Egypt	Caucasian	Mixed	Case–control	100/100	PCR-RFLP	0.63	*
Barlas et al.	2008	Turkey	Caucasian	Knee OA	Case–control	157/84	PCR-RFLP	0.34	*
Rui et al.	2018	China	Asian	Knee OA	Case–control	308/404	PCR-RFLP	0.4	*

* A significant relevance between the MMP-1 polymorphism and risk of OA was picked up in this article. Abbreviations: PCR-RFLP, PCR-restriction fragment length polymorphism; TMJ OA, temporomandibular joint OA.

### Overall analysis

Overall, there was no significant association between MMP-1-1607 (rs1799750) 1G/2G promoter polymorphism and OA (2G versus 1G, OR = 1.12, 95% CI = 0.78–1.60, *P_h_*=0; 1G/2G versus 1G/1G: OR  = 0.73, 95% CI = 0.32–1.67, *P_h_*=0; 2G/2G versus 1G/1G: OR  =  1.31, 95% CI = 0.57–2.98, *P_h_*=0; the recessive model: OR  =   1.23, 95% CI = 0.63–2.41, *P_h_*=0.57; and the dominant model: OR  =  1.25, 95% CI = 0.79–1.97, *P_h_*=0.001). The above meta-analysis results are shown in Table 2.

**Table 2:** Meta-analysis for MMP-1-1607 polymorphism and OA risk

Variables	*n*	2G vs. 1G		2G/2G + 2G/1G vs. 1G/1G		2G/2G vs. 1G/2G + 1G/1G		1G/2G vs. 2G/2G		2G/2G vs. 1G/1G	
		OR (95% CI)	*P_*h*_*	OR (95% CI)	*P_*h*_*	OR (95% CI)	*P_*h*_*	OR (95% CI)	*P_*h*_*	OR (95% CI)	*P_*h*_*
Total	6	1.12	0	1.25	0.001	1.23	0.57	0.73	0	1.31	0
		(0.78–1.60)		(0.79–1.97)		(0.63–2.41)		(0.32–1.67)		(0.57–2.98)	
Type of disease											
Knee OA	4	0.93	0	0.92	0.002	0.83	0	0.48	0	0.76	0
		(0.58–1.48)		(0.48–1.74)		(0.37–1.87)		(0.16–1.43)		(0.25**–**2.27)	
Other	2	1.63	0.008	2.03	0.192	2.74	0	1.57	0.191	3.46	0.311
		(0.83–3.19)		(1.26–3.26)		(1.80–4.16)		(0.94–2.60)		(2.13–5.62)	
Ethnicity											
Asian	3	1.16	0.014	1.31	0.054	1.58	0.002	0.45	0	1.7	0
		(0.84–1.59)		(0.84–2.04)		(0.87–2.89)		(0.13–1.94)		(0.78–3.71)	
Caucasian	3	1.07	0	1.09	0	0.94	0	1.14	0.024	0.95	0
		(0.44–2.59)		(0.35–3.34)		(0.21–4.34)		(0.45–2.61)		(0.13–6.92)	

Random-effects model was used when the *P*-value for the heterogeneity test was <0.05; otherwise, a fixed-effects model was used. Abbreviations: *n*, number of studies; *P_h_*, value of Q-test for heterogeneity test, value of Q-test for heterogeneity test.

### Stratified analysis by type of disease and ethnicity

No significant association was found in knee OA (KOA), other types of OA, Asians, or Caucasians under any genetic model when analyzing the association between MMP-1-1607 (rs1799750) and OA by ethnicity or type of disease. The primary results of subgroup analysis are presented in Table 2.

### Sensitivity analysis and publication bias

**Figure 2:**
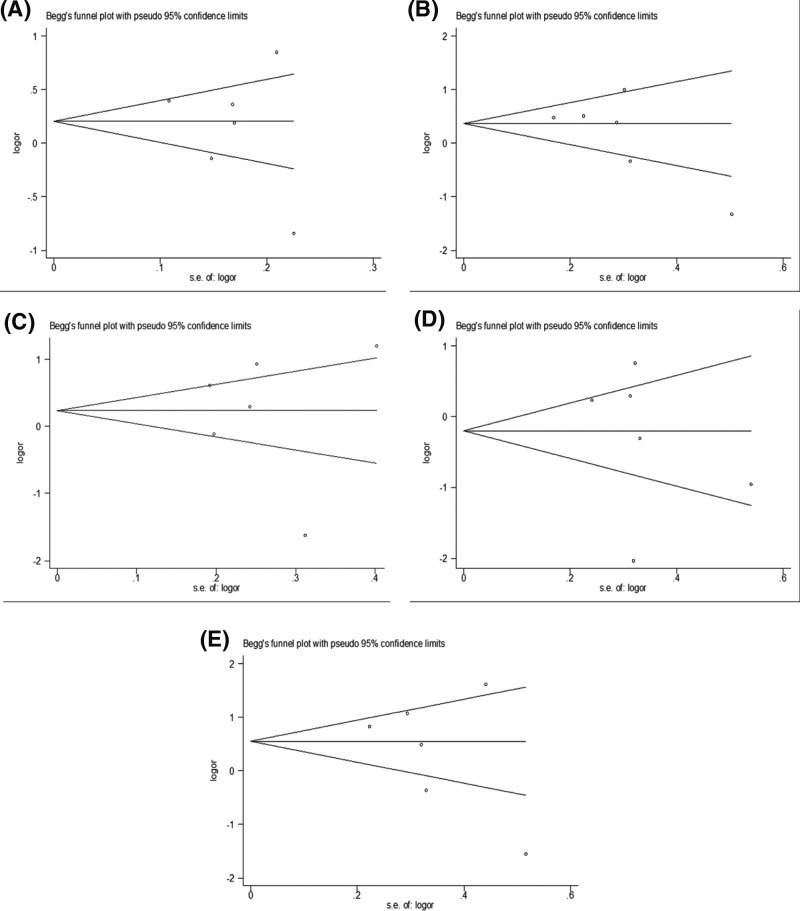
Funnel plots of the association betwen MMP-1-1607 (rs1799750) 1G/2G polymorphism and OA (**A**) Alleles model: 2G versus 1G; (**B**) Dominant model: 2G/2G+1G/2G versus 1G/1G; (**C**) Recessive model: 2G/2G versus 1G/2G + 1G/1G; (**D**) Alleles model: 1G/2G versus 2G/2G; (**E**) Alleles model: 2G/2G versus 1G/1G .

Sensitivity analysis was utilized to estimate the stability of the results. The sensitivity analysis procedure involved a single study in the meta-analysis being deleted one at a time, but the results did not change. The form of the Begg’s funnel plot did not show any evidence of apparent asymmetry in all genetic models (Figure 2). Furthermore, the Egger’s regression analysis did not reveal any evidence of publication bias (*P* =0.707 for 2G versus 1G, *P*= 0.452 for 2G/2G versus 1G/1G, *P*=0.452 for 1G/2G versus 1G/1G, *P*=0.851 for the recessive model, and *P* = 0.133 for the dominant model, respectively).

## Discussion

Since 2008, six studies have focused on the relationship between the SNP at position -1607 of MMP-1 and OA susceptibility. Barlas et al. [18] found 1G/1G carriers are susceptible to OA in Turkey, and Abd-Allah et al.’s [16] results of MMP1-1607 (rs1799750) 1G/2G polymorphism in OA were in agreement with the results of Barlas et al. [18]. However, Rui et al. [20] found 2G allele and 2G/2G carriers have an association between MMP-1-1607 (rs1799750) 1G/2G and susceptibility to OA. However, other studies have reported negative results [15,19]. All in all, the relationship between MMP-1-1607 (rs1799750) 1G/2G polymorphism and susceptibility to OA is controversial. According to one study, MMP-1 is associated with various specific pathological states, including inflammation, immunity, tumor invasion, and metastasis [21]. However, there is considerable controversy over the association between MMP-1 gene polymorphism and OA. In essence, OA is the most common cause of synovial joint tissue inflammation, leading to the progressive destruction of cartilage and bone [22].

To date, this article is the most recent and includes the first meta-analysis to determine if there is an association between MMP-1 gene polymorphism and OA. The results show that there was no association between MMP-1-1607 (rs1799750) 1G/2G polymorphism and OA susceptibility. Neither allele frequency nor genotype distribution were significantly associated with susceptibility to OA. Considering that ethnicity may influence the results, subgroup analyses were performed to further investigate the potential association. However, similar results were observed in Caucasians and KOA.

In our meta-analysis study, we did not explore any relationship of MMP-1 polymorphism with susceptibility to OA for several reasons. First, OA is a complex and multifactorial disease and includes joint injury, aging, obesity, and heredity, which lead to the progression of OA [23,24]. Second, some other unidentified genes might conceal the influence of the alleles. Third, OA is sex-associated disease [15,25]. Hence, our results imply that further investigations are required before we are able to determine the association between the MMP-1-1607 (rs1799750) 1G/2G polymorphism and OA.

In addition, some limitations of this current meta-analysis should be mentioned. First, study heterogeneity existed in all comparisons, which may affect the precision of results despite using a random-effects model to pool ORs. We believe this heterogeneity may result from the confounding factors, such as case definition, gender, and sample size. Although we carried out sensitivity analysis, obvious heterogeneity was still observed. Second, data included Caucasians and Asians which should have been optimized by gender and population in this meta-analysis. In addition, the Begg’s funnel plots and Egger’s regression statistic method both showed no obvious publication bias in this meta-analysis.

## Conclusion

We obtained strong evidence that MMP-1-1607 (rs1799750) gene polymorphism plays no role in the risk of OA, which is an important breakthrough. However, our results were obtained using a limited sample size, and therefore, this is a preliminary conclusion. Validation using more multicenter case–control studies from diverse ethnic populations is needed to confirm our findings.

## Abbreviations

CI: confidence interval
HWE: Hardy–Weinberg equilibrium
KOA: knee osteoarthritis
MMP: matrix metalloproteinase
NOS: Newcastle–Ottawa Scale
OA: osteoarthritis
OR: odds ratio
SNP: single nucleotide polymorphism
